# Bile acids destabilise HIF-1α and promote anti-tumour phenotypes in cancer cell models

**DOI:** 10.1186/s12885-016-2528-2

**Published:** 2016-07-14

**Authors:** J. P. Phelan, F. J. Reen, N. Dunphy, R. O’Connor, F. O’Gara

**Affiliations:** BIOMERIT Research Centre, School of Microbiology, University College Cork - National University of Ireland, Cork, Ireland; School of Biochemistry and Cell Biology, University College Cork - National University of Ireland, Cork, Ireland; School of Biomedical Sciences, Curtin Health Innovation Research Institute, Curtin University, Perth, WA 6102 Australia

**Keywords:** Bile acids, HIF-1 transcription factor, HIF-1α subunit, Cancer models, Hypoxia, Dimethyloxaloglycine (DMOG)

## Abstract

**Background:**

The role of the microbiome has become synonymous with human health and disease. Bile acids, as essential components of the microbiome, have gained sustained credibility as potential modulators of cancer progression in several disease models. At physiological concentrations, bile acids appear to influence cancer phenotypes, although conflicting data surrounds their precise physiological mechanism of action. Previously, we demonstrated bile acids destabilised the HIF-1α subunit of the Hypoxic-Inducible Factor-1 (HIF-1) transcription factor. HIF-1 overexpression is an early biomarker of tumour metastasis and is associated with tumour resistance to conventional therapies, and poor prognosis in a range of different cancers.

**Methods:**

Here we investigated the effects of bile acids on the cancer growth and migratory potential of cell lines where HIF-1α is known to be active under hypoxic conditions. HIF-1α status was investigated in A-549 lung, DU-145 prostate and MCF-7 breast cancer cell lines exposed to bile acids (CDCA and DCA). Cell adhesion, invasion, migration was assessed in DU-145 cells while clonogenic growth was assessed in all cell lines.

**Results:**

Intracellular HIF-1α was destabilised in the presence of bile acids in all cell lines tested. Bile acids were not cytotoxic but exhibited greatly reduced clonogenic potential in two out of three cell lines. In the migratory prostate cancer cell line DU-145, bile acids impaired cell adhesion, migration and invasion. CDCA and DCA destabilised HIF-1α in all cells and significantly suppressed key cancer progression associated phenotypes; clonogenic growth, invasion and migration in DU-145 cells.

**Conclusions:**

These findings suggest previously unobserved roles for bile acids as physiologically relevant molecules targeting hypoxic tumour progression.

## Background

The generation of intratumoral hypoxia (oxygen limitation) is a steadfast predictor of tumour fate in cancer progression and metastasis. Hypoxic cells acquire intrinsic molecular characteristics leading to therapy resistance and aggressiveness [1]. Prevalent in many cancers, (e.g. breast, cervix, ovary, prostate and the uterus), hypoxic micro-environments are associated with oxygen mediated molecular complexity, dynamic vascular alterations and increased mortality rates [2, 3]. The molecular challenges are therefore complex; the identification of hypoxic triggers and the development and exploration of innovative strategies to combat the aggressive hypoxic environment.

Recently, several studies have suggested that bile acids (BAs) and synthetic bile acid derivatives, at physiological concentrations, exhibit inhibitory effects on the in vitro cellular development and progression of several cancers including, prostate cancer [4], gastric carcinoma [5] and colon cancer [6]. BAs control cholesterol homeostasis, and have been likened to central signalling molecules relaying ingestion and energy availability messages to peripheral tissues [7, 8]. Found in the liver, primary BAs (cholic acid (CA), chenodeoxycholic acid (CDCA) are synthesised from cholesterol [9]. These molecules are metabolised by bacteria in the gastrointestinal (GI) tract producing secondary BAs (deoxycholic acid (DCA) and lithocholic acid (LCA). BAs are extensively recycled (~95 %) via the enterohepatic circulation (EHC) to maintain BA equilibrium in the gut [7].

Dysregulation of BA metabolism by infection, diet, stress or inflammation leads to dysbiosis, which is associated with several debilitating disease conditions such as Crohn’s disease (CD), Coeliac disease, Inflammatory Bowel Disease (IBD), atherosclerosis, hypercholesterolemia and gastrointestinal (GI) disease [7, 10]. While diet and genetics play roles in dysregulation and perturbations, and gut microflora composition is important for disease transition, there is growing evidence that BAs are implicated in cancer progression. Several studies have demonstrated the anti-proliferative and anti-invasive effects of BAs in different cancer cell models [4–6]. Not limited to phenotypic characterisation, molecular evidence suggests that the nuclear bile acid receptor, FXRα and the membrane bound G-protein coupled receptor TGR5 play significant roles in linking BAs and digestion to cancer progression [8]. These studies suggest hitherto unexplored links between the microbiome, the diet and cancer aetiology [11, 12].

HIF-1 (full length protein comprising HIF-1α and HIF-1β subunits) is a master transcriptional regulator implicated in several cancers, where overexpression during hypoxia is associated with tumour aggression, invasiveness, and resistance to conventional therapies in cervical, breast, oligodendroglioma, oropharyngeal, ovarian, endometrial cancer and gastro-intestinal stromal tumours of the stomach [2, 13–15]. Targeting HIF-1α (HIF-1 subunit) activity in a systematic and strategic manner is essential if tumour based angiogenic, glycolytic, and intra-tumour cell functions are to be inhibited. Similarly, the blocking of HIF-1α related functions within the tumour microenvironment using innovative molecular therapies means new combinatorial approaches can be adopted, incorporating existing chemo- and radiotherapy regimens [16].

In previous work, we demonstrated BAs destabilised HIF-1α not only in IB3-1 and S9 lung epithelial cells, but also in the adenocarcinoma human alveolar basal epithelial cell line (A-549) [17]. This led us to hypothesise that BA mediated HIF-1α destabilisation may occur in other cancer cell lines. Here we tested this in A-549, MCF-7 and DU-145 cells and further tested the effects on a range of cancer phenotypes.

## Methods

### Cell lines and maintenance

Cell lines were acquired from the American Tissue Culture Collection (ATCC) (LGC Standards, Middlesex, UK). Human prostate carcinoma cells, DU-145 (ATCC-HTB-81), human lung adenocarcinoma cells, A-549 (ATCC-CCL-185) and human mammary epithelial adenocarcinoma cells, MCF-7 (ATCC-HTB-22) were maintained in basic Dulbecco’s modified Eagle’s medium (Gibco-BRL, Paisley, UK) supplemented with 10 % foetal calf serum (FCS) and 1 % (vol/vol) Penicillin/Streptomycin (both from Sigma, Ireland) (complete medium). Depending on cell type, supplements were added according to ATCC instructions. Cells were maintained in 5 % CO_2_ in a humidified atmosphere at 37 °C and were routinely seeded into T75 tissue culture flasks (Sarstedt, Ireland) and grown to a maximum of 80–90 % confluence. At seeding and prior to experiments, cells were washed in Phosphate Buffered Saline pH 7.4 (PBS) to remove cellular debris and dead cells. Cells were detached using 0.5 % Trypsin-EDTA (Sigma-Aldrich) for 5 min, counted on the Countess Cell Counter (Life Technologies, Paisley, UK). Passage numbers did not exceed 25. For all experiments (except untreated cells), cells were supplemented with 200 μm dimethyloxaloglycine (DMOG).

### Bile acid preparation

Dihydroxylated bile acids, DCA and CDCA (Sigma) were solubilised in sterile distilled water to a concentration of 50 mM. For all procedures, a final concentration of 100 μM in complete medium was used.

### HIF-1α immunofluorescence

The intracellular HIF-1α staining of cells grown on coverslips in 6 well plates was carried out using the mouse monoclonal antibody for the HIF-1α subunit (Abcam ab16066). Cells were fixed with 4 % formaldehyde in PBS pH 7.4 and incubated with a 1:20 dilution of the monoclonal antibody overnight at 4 °C. The next day, cells were washed with PBS and incubated with a DyLight-488 conjugated secondary antibody. All images were captured on a Zeiss LSM5 using the Zeiss HBO-100 microscope illuminating system. Images were processed using the Zen AIM application imaging program and converted to JPGs using Axiovision 40 Ver. 4.6.3.0. At least three biological repetitions were carried out.

### HIF-1α subunit ELISA

Cells were seeded onto 6 well plates in complete medium and incubated in 5 % CO_2_ in a humidified atmosphere at 37 °C for 1–2 days. BAs were added in fresh medium and cells re-cultured for a further 24 h. After this period, cells were scraped into 500 μl chilled PBS. Intracellular HIF-1α activity was assayed using the HIF-1α subunit Human SimpleStep ELISA kit (Abcam) according to manufacturer’s instructions with plates assayed on a 96-well plate reader at 450 nm.

### Cytotoxicity

Lactate dehydrogenase (LDH) release was assayed as a measure of cytotoxicity using an LDH colorimetric kit (Roche) according to manufacturers’ instructions. Briefly, cells were seeded onto 96 well plates and treated with 0.1 % Triton X-100 (control) or 100 μM BAs. Following a 16 h incubation at 37 °C and 5 % CO_2_, supernatants were removed and added to catalyst reaction mixture in a fresh plate and further incubated at 37 °C and 5 % CO_2_ for 30 min to allow for colour development which was quantified on a spectrophotometer plate reader at 490 nm. Cytotoxicity was expressed as a percentage of cells treated with 0.1 % Triton (100 % cytotoxicity). All assays were carried out in triplicate.

### Phase contrast microscopy

Cells were seeded into 6 well plates on sterile coverslips (Sarstedt) and treated as above for 16 h at 37 °C and 5 % CO_2_. Coverslips were removed and fixed with 4 % paraformaldehyde for 15 min, washed and stained with 0.1 % crystal violet (Sigma) for 15 min followed by rinsing in water several times and allowing the coverslips to dry. Once dry, coverslips were viewed on a BX21 Olympus microscope and images collected using the Cell’B program and processed using Adobe Photoshop.

### Annexin V apoptosis assay

Assays were carried out according to manufacturers’ (Invitrogen, UK) instructions. Briefly, cells were grown on coverslips in 6 well plates for 1–2 days under experimental conditions. Cinnabinaric acid (positive control) was added to cells at a final concentration of 150 μm. Cold PBS pH 7.4 was added to wells to wash away debris, after which Annexin V binding buffer was added to wash cells (10 mM HEPES, 140 mM NaCl, 2.5 mM CaCl_2_ pH 7.4). 15 μl Annexin V conjugate was added per 100 μl Annexin binding buffer along with 0.1 μM 4′,6-Diamidine-2′-phenylindole dihydrochloride (DAPI) for nucleus visualisation. Cells were incubated for 15 min at room temperature after which coverslips were mounted on glass slides and viewed. All images were captured on a Zeiss LSM5 using the Zeiss HBO-100 microscope illuminating system. Images were processed using the Zen AIM application imaging program and converted to JPGs using Axiovision 40 Ver. 4.6.3.0. At least three biological repetitions were carried out.

### Cell adhesion

96 well plates (Sarstedt) were coated with either 15 μg/ml collagen (Sigma) or Matrigel 120 μg/ml (Corning) and stored at 4 °C until required. On the day of assay, plates were equilibrated in at 5 % CO_2_ in a humidified atmosphere at 37 °C for 2 h prior to use. Newly passage cells in 100 μl aliquots were seeded onto plates in the presence or absence of BAs and left for 24 h at 5 % CO_2_ in a humidified atmosphere at 37 °C hours to adhere. Matrix free wells acted as controls. Following adhesion, cells were washed with PBS × 3 and fixed with 100 μl methanol at −20 °C for 5 min. Once removed, cells were stained for 15 min with 0.1 % crystal violet after which they were rinsed with tap water. Following 10 min air-drying, 50 μl of 0.5 % Triton X-100 was added to wells and left overnight at room temperature with gentle agitation. Plates were assayed on a plate reader at 590 nm. All assays were carried out in triplicate.

### Cell invasion

Twenty-four-well inserts with an 8 μm pore size polyethylene terephthalate membranes (Corning) were used to assess cell migration of cells using the extracellular matrix, Matrigel (120 μg/ml) which was pipetted onto the membrane. Once set, transwells were either stored at 4 °C until required or equilibrated for 2 h with media in 5 % CO_2_ in a humidified atmosphere at 37 °C prior to use. The lower chambers were filled with complete media acting as a chemoattractant. Cells were seeded in each transwell and allowed to migrate over a 24 h period in 5 % CO_2_ in a humidified atmosphere at 37 °C. Cells were fixed by replacing culture medium at the top and bottom of the transwell with 4 % formaldehyde in PBS pH 7.4 for 15 min. Once fixed, transwells were washed in PBS, stained with 0.2 % crystal violet for 10 min and washed several times in distilled water. Non-migrated cells were wiped from the transwell top using cotton buds leaving only migrated cells at the bottom of the membrane. These cells were counted using a stereomicroscope and plotted as the percentage of invading cells of the total number of plated cells. All assays were carried out in triplicate.

### Transwell migration

Twenty-four-well inserts with an 8 μm pore size polyethylene terephthalate membranes (Corning) were used to assess cell migration of cells. The lower chambers were filled with complete media acting as a chemoattractant. Cells were seeded into each transwell insert and allowed to migrate over a 24 h period in 5 % CO_2_ in a humidified atmosphere at 37 °C. Cells were fixed by replacing culture media at the top and bottom of the transwell with 4 % formaldehyde in PBS pH 7.4 for 15 min. Once fixed, transwells were washed in PBS, stained with 0.2 % crystal violet for 10 min and washed several times in distilled water. Non migrated cells were carefully wiped away from the top of the transwell using cotton buds leaving only migrated cells at the bottom of the membrane. These cells were counted using a stereomicroscope and plotted as the percentage of invading cells of the total number of plated cells. All assays were carried out in triplicate.

### Clonogenic assay

Cells, seeded in 6 well plates at low density in complete medium, were incubated with BAs for a period of 1–3 weeks in 5 % CO_2_ in a humidified atmosphere at 37 °C. Plates were left undisturbed for this period. After 3 weeks, medium was removed and cells rinsed with PBS. Next, cells were incubated in 4 % formaldehyde in PBS pH 7.4 for 20 min after which they were rinsed in PBS and incubated with 0.5 % crystal violet (Sigma, Ireland) for 2 h at room temperature. Wells were rinsed in tap water to remove crystal violet and left to air dry overnight. Once dry, colonies were counted using a stereomicroscope to determine plating efficiencies (PE) and survival fractions (SF). PE = no. of colonies formed/no. of cells seeded × 100 %. SF = no. of colonies formed after treatment/no. of colonies seeded × PE. All experiments were carried out in triplicate.

### Wound healing migration assay

Cells were plated into individual round dishes (Corning) and grown to 80–90 % confluency for 2–4 days in 5 % CO_2_ in a humidified atmosphere at 37 °C in serum free media. Once confluent, a “wound” was generated across the cell lawn using a sterile blue tip after which media was removed and replaced with media containing BAs. Time zero dishes were prepared along with 24 h dishes to assess closing of the wound relative to time. After this period, dishes were fixed with 4 % formaldehyde in PBS pH 7.4 for 15 min. Once fixed, dishes were washed in PBS, stained with 0.2 % crystal violet for 10 min and washed several times in distilled water. All dishes were scored for migration distances on a stereomicroscope and distances migrated between “wound” viewed and captured on an Olympus BX-51.

### Metabolic activity assay

DU-145 cells (untreated, DMOG treated and BAs + DMOG treated cells) were assessed for metabolic activity using the XTT (2,3-Bis-(2-Methoxy-4-Nitro-5-Sulfophenyl)-2*H*-Tetrazolium-5-Carboxanilide) assay at 24 h intervals (over 4 days) in 96 well plates in 5 % CO_2_ in a humidified atmosphere at 37 °C. Briefly, 100 μl freshly prepared XTT (Sigma) and menandione was added to cells in 96 well plates. After a 2 h incubation, cell metabolic activity was determined by measuring the absorbance at 490 nm on a microplate spectrophotometer. All assays were performed in triplicate.

### Proliferation assay

DU-145 cells (untreated, DMOG treated and BAs + DMOG treated cells) were seeded into 24 well plates and assessed for proliferation over a 15 day period. For staining, culture media was aspirated and cells fixed with 4 % paraformaldehyde at room temperature for 10 min. Cells were then stained with 0.05 % crystal violet for 30 min after which cells were washed in water and air dried on filter paper. Crystal violet dye was dissolved in 500 μl methanol and emission spectra measured at a wavelength of 595 nm on a microplate spectrophotometer. All assays were performed in triplicate.

### Viability assay

DU-145 cells (untreated, DMOG treated and BAs + DMOG treated cells) were assessed for cell viability using trypan blue (live/dead) staining. Cells were seeded into T25 flasks and assessed for cell viability every 24 h over a 5 day period. On the day of assay, cells were gently scraped from the flask surface and 2 ml of PBS added. The volume was mixed thoroughly (to remove clumps) and centrifuged at 1200 rpm for 2 min to pellet cells. The supernatant was decanted and the pellet suspension mixed. Cells were removed and added to trypan blue and mixed (to remove clumps) thoroughly. An aliquot was loaded onto a disposable chamber slide and placed into the Countess™ Automated Cell Counter (Invitrogen) to count live/dead cells (viability), represented as percentage cell viability. All assays were performed in triplicate. Random counting of cells was also carried out using a haemocytometer and inverted microscope to assess the validity of Automated Cell Counter data. All visual counts were within +/- 5 % of Cell Counter data (data not shown).

### Real-Time PCR

DU-145 cells (untreated, DMOG treated and BA + DMOG treated) were collected in RNA Protect Cell Reagent (Qiagen, West Sussex, UK) and stored at −80 °C until RNA extraction, using the RNeasy Minikit as per the manufacturer’s instructions (Qiagen). DNase treatment and reverse transcription were both performed using the Quantitect Reverse Transcription kit (Qiagen). Approximately 1 ug of RNA was reverse transcribed into cDNA as per protocol. Quantitative RT-PCR was performed using the FastStart Taqman probe master mix (Roche) on a PTC-200 thermocycler (Bio-Rad) according to manufacturer’s instructions. Primers were designed using the Universal Probe Library Assay Design Centre (Roche). HPRT-1 (Forward); gaccagtcaacaggggacat, HPRT-1 (Reverse); gtgtcaattatatcttccacaatcaag (Probe 22). HK II (Forward); tcccctgccaccagacta, HK II (Reverse); tggacttgaatcccttggtc (Probe 54).

### Statistical analysis

Three independent biological replicates (*N* = 3) were performed for each experiment unless otherwise stated. Statistical significance was measured using a paired two-tailed Student’s *T*-test. Differences were considered to be statistically significant if the *p*-value were; * *P* < 0.05; ** *P* < 0.01.

## Results

### Dihydroxylated BAs destabilise HIF-1α in A-549, MCF-7 and DU-145 cells

**Fig. 1:**
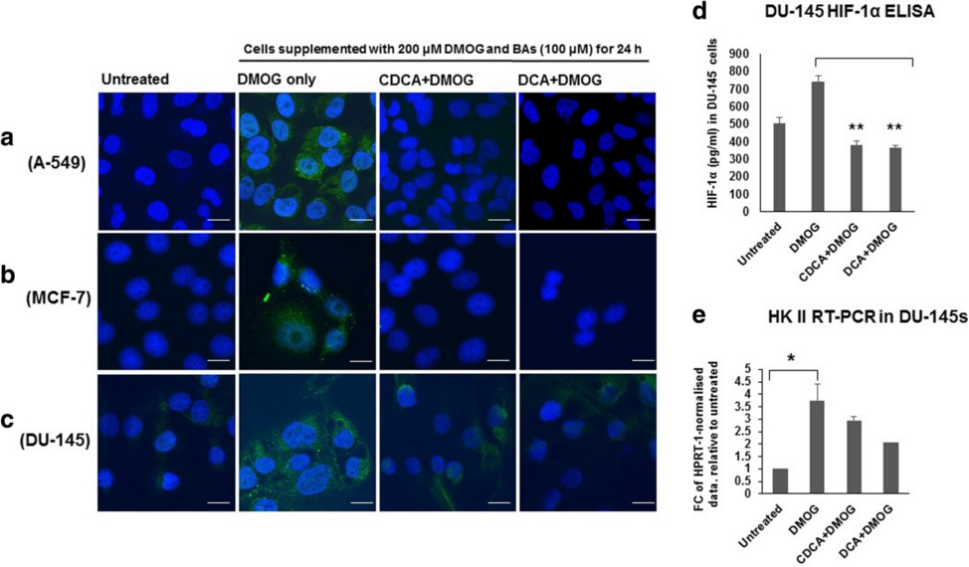
Dihydroxylated bile acids CDCA and DCA destabilise HIF-1α in A-549, MCF-7 and DU-145 cells. BAs destabilise HIF-1α in (**a**) A-549 cells, (**b**) MCF-7 cells and (**c**) DU-145 cells. For fluorescent microscopy, all cells were grown under hypoxic conditions (DMOG 200 μM), except for untreated cells. All cells were stained for intracellular HIF-1α (*green*) using an anti-HIF1α antibody and 4′, 6-diamidino-2-phenylindole (DAPI) for DNA nuclear material (*blue*). HIF-1α staining was not observed in untreated cells whereas in DMOG treated cells, HIF-1α punctate staining was observed. Scale bar = 20 μM, magnification × 63 oil immersion. HIF-1α was quantitatively assayed in DU-145 cells using an anti-HIF-1α ELISA in the presence of BAs (**d**). Untreated cells exhibited reduced HIF-1α expression when compared to DMOG treated cells whereas in BA treated cells, HIF-1α levels were lower. **e**. Hexokinase II RT-PCR. Expression data normalised to HPRT is the average of two biological replicates (with two technical replicates) and is presented as fold change relative to the untreated control. Statistical analysis was performed using Repeated Measures ANOVA with post-hoc Bonferroni testing (* *p* < 0.05). For other data, statistical significance was assessed via an unpaired, two-tailed Student *t* tests. *, *P <* 0.05. Data presented represent those from three independent replicates

We previously demonstrated that BAs induced destabilisation of HIF-1α not only in IB3-1 and S-9 epithelial cells but in A-549 lung adenocarcinoma cells [17]. Here, we assessed HIF-1α destabilisation in three cancer cell lines using two independent methods; immunofluorescence with an anti-HIF-1α antibody in A-549, MCF-7 and DU-145 cells (Fig. 1a-c) and an anti-HIF-1α subunit ELISA in DU-145 cells (Fig. 1d). Untreated cells (normoxic conditions) were devoid of green fluorescent punctae suggesting low HIF-1α levels that would indicate rapid turnover of HIF-1α under these conditions. DMOG treated (hypoxia mimetic conditions) cells exhibited widespread HIF-1α punctate staining indicative of stabilised HIF-1α expression as expected. In cells treated with DMOG plus either DCA or CDCA, HIF-1α detection was faint and dispersed in DU-145 cells (Fig. 1c), while in A-549 and MCF-7 cells (Fig. 1a and b), no HIF-1α staining was observed. This suggests a reduction in HIF-1α expression and implicates BAs in HIF-1α destabilisation. We also assessed HIF-1α quantitatively in DU-145 cells using an anti-HIF-1α ELISA (Fig. 1d). As expected HIF-1α expression was increased in DMOG treated cells compared to untreated controls. The presence of BAs reduced HIF-1α expression in DMOG treated cells. This supports the conclusion that BAs generally promote HIF-1α destabilisation under hypoxic conditions. Finally, we investigated the expression of hexokinase II (HK II), a downstream transcriptional target of HIF-1α (Fig. 1e). These data showed that HK II expression increased in DMOG treated cells, whereas for both BAs, HK II expression in DMOG treated cells was not statistically different to the untreated control.

### Dihydroxylated BAs are not cytotoxic towards A-549, DU-145 and MCF-7 cancer cells

**Fig. 2:**
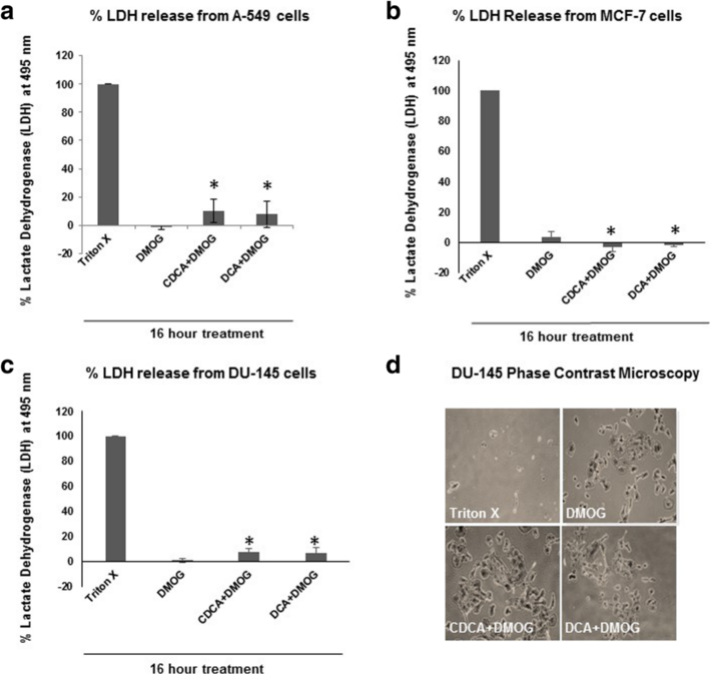
Dihydroxylated bile acids CDCA and DCA are not cytotoxic towards A-549, MCF-7 and DU-145 cells. For cytotoxicity assays, all cells were grown under hypoxic conditions (DMOG 200 μM), except for untreated cells. Cells in the presence of 100 μM dihydroxylated BAs (CDCA and DCA) were grown for 16 h and assessed for lactate dehydrogenase (LDH) production. Cytotoxicity was expressed as the mean percentage standard deviation (SD) (*n =* 3) of the total LDH released from cells treated with 0.1 % Triton X-100, which was given an arbitrary value of 100 %. A-549 cells (**a**), MCF-7 cells (**b**) and DU-145 cells (**c**) exhibited minimal cytotoxicity in the presence of BAs. DU-145 morphology in the presence of BAs was assessed by phase contrast microscopy (**d**). Cells incubated with BAs exhibited cell shape and morphology similar to untreated cells. Data presented represent those from three independent replicates. Student T-tests were performed for comparison of treated cells with control untreated cells (* *P <* 0.05)

Before assessing the impact of BAs on cancer cell phenotypes, it was important to establish whether or not BAs exhibited cytotoxic activity towards these cells. The effects of dihydroxylated BAs was assessed in all cell lines using a lactate dehydrogenase (LDH) cytotoxicity assay (Fig. 2a-c). Compared with exposure of cells to 0.1 % Triton X-100 that was assigned an arbitrary 100 % LDH release value, untreated cells released minimal LDH levels, reflective of uncompromised plasma membranes and retained intracellular LDH. In the presence of BAs, all cell lines released negligible (<10 %) LDH reflecting the minimal impact of BAs on cytotoxicity at this time-point.

We also assessed the impact of BAs on cell morphology using simply phase contrast microscopy (shown for DU-145 in Fig. 2d). This indicated no obvious differences between untreated and BA treated cells. Overall, these assays indicated that minimal cytotoxic effects were observed upon short-term exposure to BAs, which is in the time frame in which motility and invasion assays can be performed.

### BAs reduce DU-145 cell adhesive, migratory and invasive potential

The DU-145 cell line was chosen as a cell model in which to establish the impact of bile acids on cell phenotypes associated with metastasis, including adhesion, invasion and migratory capacity. DU-145 cells are derived from an androgen-independent prostate cancer and represents an aggressive cancer cell phenotype that has undergone an Epithelial Mesenchymal Transition (EMT) that can be reversed by suppressing regulators of EMT pathways in vitro [18].

**Fig. 3:**
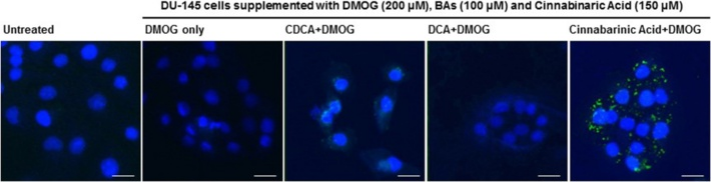
Dihydroxylated BAs do not induce apoptosis in DU-145 cells. For apoptosis assays, all cells were grown under hypoxic conditions (DMOG 200 μM), except for untreated cells. DU-145 cells in the presence of 100 μM dihydroxylated BAs (CDCA and DCA) were grown for 16 h and stained with 4′,6-diamidino-2-phenylindole (DAPI) for deoxyribonucleic acid (DNA) (*blue*) and Annexin V AlexaFluor 488 fluorescence (*green*). Supplementation of the red pigmented phenoxazinone, cinnabarinic acid (150 μM) to cells acted as positive control. Untreated cells and cells treated with BAs displayed no green fluorescent punctae, whereas positive control cells exhibited green fluorescent punctae indicative of apoptosis at the membrane. Fluorescent microscopy data represent those from one of three independent experiments. Between 10 and 15 images were recorded for each condition. Scale bar = 20 μM. Magnification × 63

We first established whether BAs triggered apoptosis in DU-145 cells using FITC-labelled Annexin V to detect exposed plasma membrane phosphatidylserine (PS) and DAPI nuclear stain. Untreated control cells did not present any PS staining (Fig. 3). In contrast, cells incubated with the pro-apoptotic positive control [19], cinnabinaric acid (150 μM) exhibited extensive PS staining indicating extensive apoptosis induction. BA-treated cells exhibited negligible green punctae indicating that BAs do not induce apoptosis in DU-145 cells. This assay combined with the cytotoxicity data (Fig. 2) demonstrates that short term exposure to BAs has minimal impact on cytotoxicity and apoptosis. Thus the effects of BAs on cell adhesion, migration and invasion could be further investigated without cell death confounding assays.

**Fig. 4:**
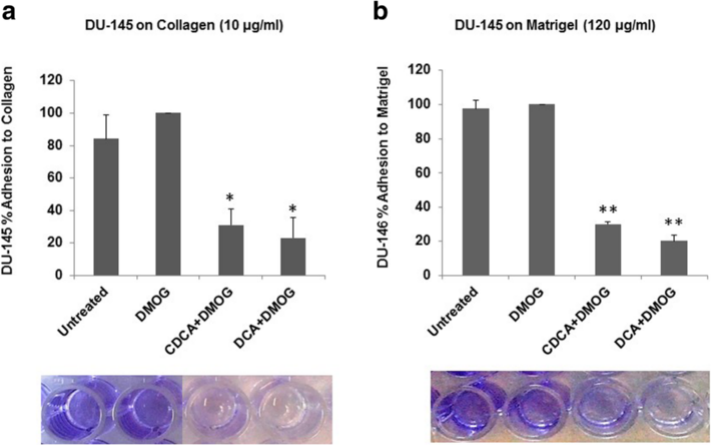
DU-145 adhesion to collagen and matrigel in the presence of dihydroxylated BAs is reduced. For adhesion assays, all cells were grown under hypoxic conditions (DMOG 200 μM), except for untreated cells. DU-145 cells in the presence of 100 μM dihydroxylated BAs were seeded into 96 well plates coated with collagen and matrigel and allowed adhere for 24 h after which supernatants were removed and adhered cells washed and stained with crystal violet. Plates were assayed on a plate reader at 550 nm. All data were derived from three independent experiments, *N* = 3, S.D; * *P* < 0.05; ** *P* < 0.01

The in vivo binding of cells to the extracellular matrix (ECM) is essential for cell survival and cell-to-cell communication, but is also essential for the metastatic potential of cancer cells [20]. The effects of BAs on cell adhesion were assessed in vitro by allowing DU-145 cells to adhere to either collagen or Matrigel in the presence of BAs and assessing the relative numbers of attached cells. Untreated and DMOG-treated cells exhibited similar adhesion to collagen (Fig. 4a) or Matrigel (Fig. 4b). Cells treated with CDCA or DCA exhibited considerably reduced cell adhesion compared to controls (Fig. 4a and b). This suggests that BAs reduce the ability of DU-145 cells to adhere to ECMs which may impact on their migration and invasion capacity.

**Fig. 5:**
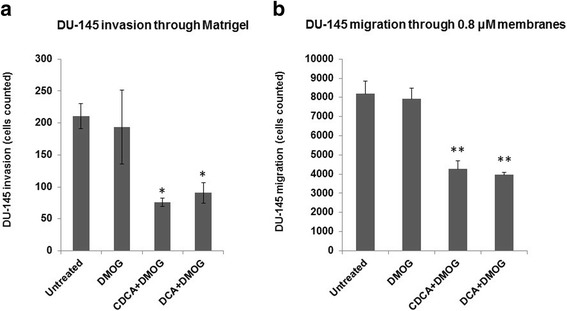
Dihydroxylated BAs impair invasion and migration of DU-145 cells. For invasion and migration assays, all cells were grown under hypoxic conditions (DMOG 200 μM), except for untreated cells. Boyden chambers alone (migration) and those containing Matrigel (invasion) were hydrated for at least 2 h with 500 μl serum free media in the bottom of the well and 500 μl serum free media in the chamber. Once hydrated, the media in the bottom was replaced with media supplemented with 10 % FCS and the media in the chamber was replaced with cells in media supplemented with 10 % FCS. After growing for 24 h at 5 % CO_2_ in a humidified atmosphere at 37 °C, cells were fixed, rinsed, dried and counted. Cell migration and invasion data were derived from three independent experiments, *N* = 3, S.D; * *P* < 0.05; ** *P* < 0.01

The in vitro invasive and migratory capacity of metastatic cancer cells provide insights into how cells behave in the presence of modulators of cancer progression. Here, the invasion capabilities of DU-145 cells in the presence of BAs were assessed using Matrigel (on 0.8 μm membranes) (Fig. 5a) and directional migration was assessed using Transwell assays (Fig. 5b). DU-145 cell invasion and migration were both reduced approximately 2-fold compared to untreated control cells. This indicates that BAs impair the ability of these cells to invade and migrate efficiently.

**Fig. 6:**
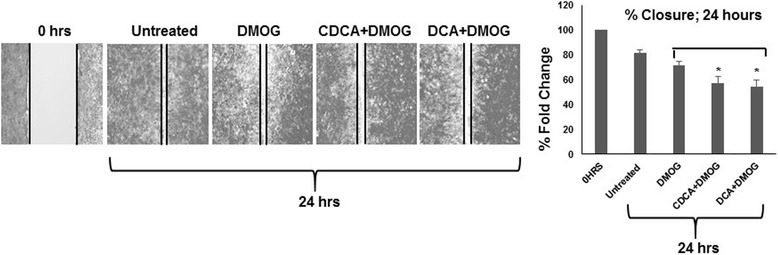
Dihydroxylated BAs impair scratch-wound healing in DU-145 cells. For scratch-wound healing assays, all cells were grown under hypoxic conditions (DMOG 200 μM), except for untreated cells. Monolayer cells were grown until 80 % confluent, wounded, stained and images recorded after wounding at 0 and 24 h. Distances between scratch-wound fronts were measured using a steromicroscope and were represented as μm. At least 20 measurement points were taken per condition and averaged data were derived from three independent experiments, *N* = 3, S.D; * *P* < 0.05; ** *P* < 0.01

To further explore migratory potential, a wound healing assay was performed (Fig. 6). One of the advantages of this assay is that it mimics the migration of wounded cells in vivo [21]. Untreated DU-145 monolayers were wounded at 0 h, fixed, stained and photographed to establish a basal reference wound. Treated cells were then allowed to fill the wound and at 24 h were similarly stained and photographed to assess cell migration relative to both time and the presence of BAs. Compared to the time 0 h reference wound (Fig. 6) both untreated and DMOG treated cells displayed extensive wound closures after 24 h, reflective of similar cell migratory patterns. Cells exposed to BAs exhibited slightly reduced wound healing patterns at 24 h for both CDCA and DCA respectively. These data demonstrate BAs impaired DU-145 cell migratory potential.

### The clonogenic potential of DU-145 and MCF-7 cells is significantly reduced in the presence of BAs

**Fig. 7:**
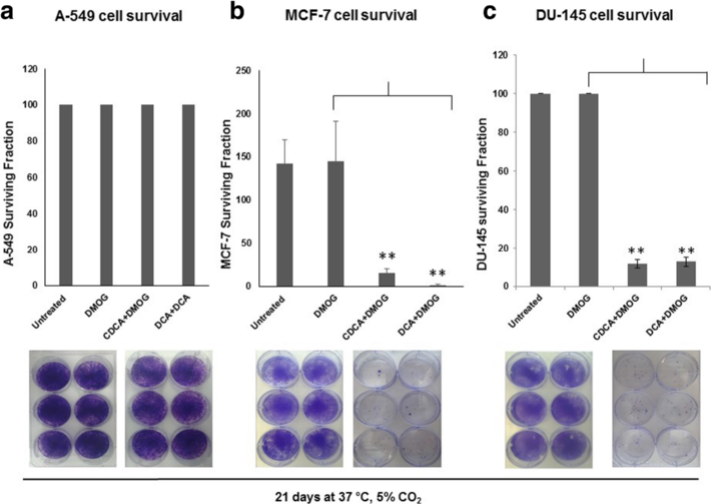
Dihydroxylated bile acids CDCA and DCA affect the clonogenic ability and cell survival of MCF-7 and DU-145 cells. For clonogenic assays, all cells were grown under hypoxic conditions (DMOG 200 μM), except for untreated cells. A-549 cells (**a**), MCF-7 cells (**b**) and DU-145 cells (**c**) in the presence of 100 μM BAs cells were seeded into 6 well plates and grown over a twenty one day period. Resulting colonies were washed, stained with 0.5 % crystal violet and counted using a steromicroscope. To calculate the surviving fraction (SF), the plating efficiency (PE) was calculated; PE = number of colonies formed/no. of cells seeded × 100 %. This value was used to determine the SF; number of colonies formed after treatment/no. of cells seeded × PE. Clongenic data were derived from three independent experiments, *N* = 3, S.D; * *P* < 0.05; ** *P* < 0.01

**Fig. 8:**
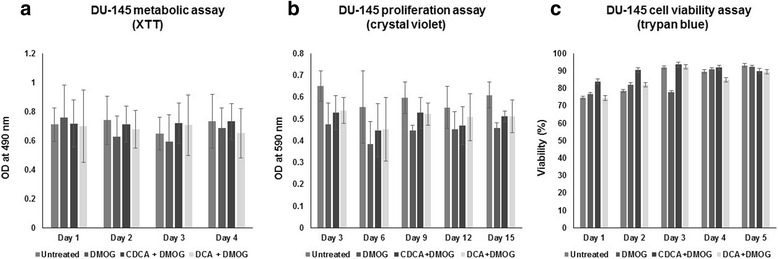
Dihydroxylated bile acids CDCA and DCA do not affect the metabolic activity, proliferation and cell viability of DU-145 cells. For metabolic activity (XTT), proliferation (*crystal violet*) and cell viability (*trypan blue*) assays, all cells were grown under hypoxic conditions (DMOG 200 μM), except for untreated cells. For all assays, no significant compromises were observed in cell metabolic activity, cell proliferation and cell viability with respect to time. All data were derived from three independent experiments

To investigate the long-term effects of exposure of cancer cells to BAs, we used clonogenic assays which measure a key cancer cell phenotype; the ability to form colonies when seeded at low density. The clonogenic potential of A-549, MCF-7 and DU-145 cells was assessed in the presence of BAs over a twenty one day period (Fig. 7a-c). The cell survival fraction (an index of clonogenic growth and cell survivability) was reduced for MCF-7 and DU-145 cells (Fig. 7b and c) but was unaltered for A-549 cells (Fig. 7a). For all cell lines tested, untreated and DMOG-treated cells were characterised by high colony density (no measurable differences). Similarly, BAs had no effect on A-549 clonogenicity as evidenced by dense staining in all plates, high colony numbers and no measurable differences. However, BA-treated DU-145 and MCF-7 cells exhibited greatly reduced colony numbers. These data demonstrate that BAs markedly inhibited the clonogenic and survival potential of DU-145 and MCF-7 cells. This suggests that exposure to BAs would limit the survival and metastatic potential of cancer cells under conditions of hypoxia. Importantly, in DU-145 cells, the reduction in colony formation numbers was independent of metabolic activity, cell proliferation and cell viability, which all remained relatively unchanged over time of exposure to BAs (Fig. 8a–c).

## Discussion and conclusions

The main characteristics of cancer cell progression are abnormal cell growth and proliferation followed by rapid migration, invasion, adhesion and the formation of tumours at new sites [22]. Concomitant to external phenotypic changes, many epigenetic changes occur within the tumour environment, most notably in hypoxic tumours where HIF-1 (full length) activation in combination with reduced oxygen and down-stream effector activation, contribute to a tumour microenvironment that favours phenotypic changes necessary for metastasis [14]. We demonstrated HIF-1α destabilisation in A-549, MCF-7 and DU-145 cells, reinforcing the premise that expression of this key transcription factor can be regulated by CDCA and DCA and suggests a novel BA/HIF-1α interplay during cancer progression.

BAs were not cytotoxic towards these cells, suggesting this destabilisation phenotype was influenced by the presence of BAs. HIF-1α subunit expression and stabilisation is a common phenomenon observed in many hypoxic tumours, therefore its strategic targeting and destabilisation is of critical value from a clinical and survival perspective [14, 23, 24]. The HIF-1α subunit may be targeted at the transcriptional, translational and post-translational levels which has allowed development of agents that destabilise or block HIF-1α activation [25]. Many chemical and molecular inhibitors have been developed to destabilise HIF-1α [26–28], however, many have not progressed to clinical trials due to non-specificity and cytotoxicity issues [29]. Our data suggests that BAs could provide an innate protection against hypoxic tumour development and circumvent specificity and cytotoxicity issues. Beneficial BAs could be supplied via the diet or from specific pools of BAs derived from ingestion of bile producing bacteria.

Not only was a HIF-1α destabilisation phenotype observed in all cancer cells tested, but BAs greatly reduced the clonogenic potential of DU-145 and MCF-7 cells. Specifically for DU-145 cells, clonogenicity was independent of metabolic activity, cell proliferation and cell viability, suggesting BAs targeted the cells reproductive ability to form progenies. Furthermore the adhesive, migratory and invasive capacity of DU-145 cells were consistently decreased by BAs. Taken together, with the decreased clonogenic growth, these data indicate that BAs can reverse key phenotypes associated with metastatic cancer cells, particularly in the context of hypoxia and HIF-1α destabilisation. The loss of hallmark cancer phenotypes has been reported in association with HIF-1α knock-down/destabilisation in several cell types (gastric cancer cells, pancreatic cancer cells and uveal melanoma cells [30–32]. While these reports implicate the HIF-1α subunit in cancer progression associated phenotypes, its precise mechanistic role in conjunction with BAs remains to be elucidated. However, expression of HK II, a cellular glycolytic enzyme and a transcriptional target of HIF-1α, was affected by BAs, demonstrating possible downstream perturbations of the HIF-1α network.

In terms of cancer progression, previous studies have shown dramatic effects of BAs on cancer progression in different cell models (stomach, duodenum, oesophagus and colon cancers) [33–37]. Animal models and in vitro cell assays using BAs at supra-physiological concentrations have been cited as causative agents in the generation of cancerous lesions at these sites. Prolonged exposure to BAs (e.g. stomach, colon and oesophagus) promote an *in situ* hyperplasic dysmorphia (cell elongation, proliferation and polarisation), and over time, cells become neoplastic leading to tumour development. While the molecular links between BA metabolism and cancer are not fully elucidated, definitive roles for BAs in cancer progression cannot be overlooked in view of the evidence presented in this study.

**Fig. 9:**
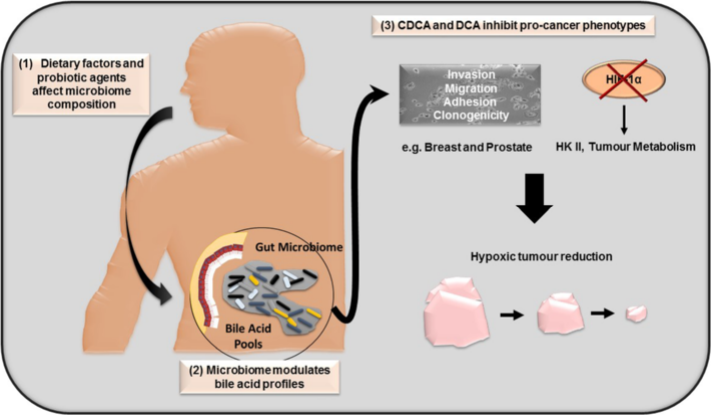
Proposed mechanism of bile acid action towards cancer progression. 1. Variations in dietary intake play a huge role in determining microbiome composition in the gut. 2. This leads to microbiome modulation of distinct bile acid profiles (CDCA and DCA). 3. Both bile acids destabilise HIF-1α, an important transcription factor involved in the hypoxic switch in tumours and target important anti-cancer phenotypes such as invasion, migration, adhesion and clonogenicity, potentially leading to hypoxic tumour reduction

Modulation of BA intake, primarily via the diet, could exert protective effects on the spread of hypoxic cancerous lesions at several sites within the body (e.g. breast and prostate) (Fig. 9). Concerted efforts to establish long-term effects of probiotics/prebiotics on dysbiosis have been proposed, however cause and effect relationships have not been established for such interventionist approaches [11, 12]. Similarly, diets high in fat, sugar and meat perturb the gut microbiota balance leading to increased risks of e.g. colorectal cancer [38]. Evidence suggests a more Mediterranean approach to diet (fruits and vegetables, whole grains, legumes and nuts, olive oil, herbs and spices, limited red meat, fish and poultry and red wine (optional) in moderation) exerts a “probable” long term protective role against cancer. However more empirical data is required, along with well designed, randomised, longitudinal studies to support these observations [39].

Bile acids exert dramatic effects on cancer development and progression. Several cancer phenotypes were significantly affected in the presence of BAs suggesting these molecules are not only important for lipid metabolism, but are potential mediators of cancer progression. Future research in this area requires extensive phenotypic characterisation of the role of BAs in other cancer models, in-depth molecular investigations of HIF-1α effectors and their specific roles in invasion, migration, adhesion and cell survival.

## Abbreviations

ATCC, American Tissue Culture Collection; BA, bile acids; CA, cholic acid; CD, Crohn’s Disease; CDCA, chenodeoxycholic acid; DAPI, 4’,6-diamidino-2-phenylindole; DCA, deoxycholic acid; DMOG, dimethyloxaloglycine; DNA, deoxyribonucleic acid; EDTA, ethylenediaminetetraacetic acid; EHC, enterohepatic circulation; ELISA, enzyme-linked immunosorbent assay; EMT, epithelial-mesenchymal transition; FC, fold change; FCS, foetal calf serum; FXR, farnesoid X receptor; GI, gastrointestinal; HIF-1α, hypoxia inducible Factor-1-alpha; HK II, hexokinase II; IBD, inflammatory bowel disease; LCA, lithocholic acid; LDH, lactate dehydrogenase; OD, optical density; PBS, phosphate buffered saline; PE, plating efficiency; PS, phosphatidylserine; SD, standard deviation; SF, survival fraction; TGR5, G protein coupled receptor; XTT, 2,3-Bis-(2-Methoxy-4-Nitro-5-Sulfophenyl)-2*H*-Tetrazolium-5-Carboxanilide

## References

[1] Brown JM, Wilson WR (2004). Exploiting tumour hypoxia in cancer treatment. Nat Rev Cancer.

[2] Semenza GL (2003). Targeting HIF-1 for cancer therapy. Nat Rev Cancer.

[3] Hoelder S, Clarke PA, Workman P (2012). Discovery of small molecule cancer drugs: successes, challenges and opportunities. Mol Oncol.

[4] Choi YH, Im EO, Suh H, Jin Y, Yoo YH, Kim ND (2003). Apoptosis and modulation of cell cycle control by synthetic derivatives of ursodeoxycholic acid and chenodeoxycholic acid in human prostate cancer cells. Cancer Lett.

[5] Pyo JS, Ko YS, Kang G, Kim DH, Kim WH, Lee BL (2015). Bile acid induces MUC2 expression and inhibits tumor invasion in gastric carcinomas. J Cancer Res Clin Oncol.

[6] Zeng H, Botnen JH, Briske-Anderson M (2010). Deoxycholic acid and selenium metabolite methylselenol exert common and distinct effects on cell cycle, apoptosis, and MAP kinase pathway in HCT116 human colon cancer cells. Nutr Cancer.

[7] Jones ML, Tomaro-Duchesneau C, Prakash S (2014). The gut microbiome, probiotics, bile acids axis, and human health. Trends Microbiol.

[8] Baptissart M, Vega A, Maqdasy S, Caira F, Baron S, Lobaccaro JM (2013). Bile acids: from digestion to cancers. Biochimie.

[9] Begley M, Hill C, Gahan CG (2006). Bile salt hydrolase activity in probiotics. Appl Environ Microbiol.

[10] Zhu Y, Michelle Luo T, Jobin C, Young HA (2011). Gut microbiota and probiotics in colon tumorigenesis. Cancer Lett.

[11] Power SE, O’Toole PW, Stanton C, Ross RP, Fitzgerald GF (2014). Intestinal microbiota, diet and health. Br J Nutr.

[12] Brown K, DeCoffe D, Molcan E, Gibson DL (2012). Diet-induced dysbiosis of the intestinal microbiota and the effects on immunity and disease. Nutrients.

[13] Diaz-Gonzalez JA, Russell J, Rouzaut A, Gil-Bazo I, Montuenga L (2005). Targeting hypoxia and angiogenesis through HIF-1alpha inhibition. Cancer Biol Ther.

[14] Zhong H, De Marzo AM, Laughner E, Lim M, Hilton DA, Zagzag D (1999). Overexpression of hypoxia-inducible factor 1alpha in common human cancers and their metastases. Cancer Res.

[15] Lau KW, Tian YM, Raval RR, Ratcliffe PJ, Pugh CW (2007). Target gene selectivity of hypoxia-inducible factor-alpha in renal cancer cells is conveyed by post-DNA-binding mechanisms. Br J Cancer.

[16] Falchook GS, Wheler JJ, Naing A, Jackson EF, Janku F, Hong D (2014). Targeting hypoxia-inducible factor-1alpha (HIF-1alpha) in combination with antiangiogenic therapy: a phase I trial of bortezomib plus bevacizumab. Oncotarget.

[17] Legendre C, Reen FJ, Woods DF, Mooij MJ, Adams C, O’Gara F (2014). Bile acids repress hypoxia-inducible factor 1 signaling and modulate the airway immune response. Infect Immun.

[18] Bowe RA, Cox OT, Ayllon V, Tresse E, Healy NC, Edmunds SJ (2014). PDLIM2 regulates transcription factor activity in epithelial-to-mesenchymal transition via the COP9 signalosome. Mol Biol Cell.

[19] Hiramatsu R, Hara T, Akimoto H, Takikawa O, Kawabe T, Isobe K (2008). Cinnabarinic acid generated from 3-hydroxyanthranilic acid strongly induces apoptosis in thymocytes through the generation of reactive oxygen species and the induction of caspase. J Cell Biochem.

[20] Lu P, Weaver VM, Werb Z (2012). The extracellular matrix: a dynamic niche in cancer progression. J Cell Biol.

[21] Rodriguez LG, Wu X, Guan JL (2005). Wound-healing assay. Methods Mol Biol.

[22] Han T, Kang D, Ji D, Wang X, Zhan W, Fu M (2013). How does cancer cell metabolism affect tumor migration and invasion?. Cell Adhes Migr.

[23] Hong SS, Lee H, Kim KW (2004). HIF-1alpha: a valid therapeutic target for tumor therapy. Cancer Res Treat.

[24] Yasuda Y, Arakawa T, Nawata Y, Shimada S, Oishi S, Fujii N (2015). Design, synthesis, and structure-activity relationships of 1-ethylpyrazole-3-carboxamide compounds as novel hypoxia-inducible factor (HIF)-1 inhibitors. Bioorg Med Chem.

[25] Lu Y, Madu C, Masters J, Lu A, Li L (2014). Development of a Novel Anti-HIF-1alpha Screening System Coupled with Biochemical and Biological Validation for Rapidly Selecting Potent Anti-Cancer Compounds. J Cancer.

[26] Lee K, Qian DZ, Rey S, Wei H, Liu JO, Semenza GL (2009). Anthracycline chemotherapy inhibits HIF-1 transcriptional activity and tumor-induced mobilization of circulating angiogenic cells. Proc Natl Acad Sci U S A.

[27] Isaacs JS, Jung YJ, Mimnaugh EG, Martinez A, Cuttitta F, Neckers LM (2002). Hsp90 regulates a von Hippel Lindau-independent hypoxia-inducible factor-1 alpha-degradative pathway. J Biol Chem.

[28] Isaacs JS, Jung YJ, Neckers L (2004). Aryl hydrocarbon nuclear translocator (ARNT) promotes oxygen-independent stabilization of hypoxia-inducible factor-1alpha by modulating an Hsp90-dependent regulatory pathway. J Biol Chem.

[29] Hu Y, Liu J, Huang H (2013). Recent agents targeting HIF-1alpha for cancer therapy. J Cell Biochem.

[30] Rohwer N, Lobitz S, Daskalow K, Jons T, Vieth M, Schlag PM (2009). HIF-1alpha determines the metastatic potential of gastric cancer cells. Br J Cancer.

[31] Shi CY, Fan Y, Liu B, Lou WH (2013). HIF1 contributes to hypoxia-induced pancreatic cancer cells invasion via promoting QSOX1 expression. Cell Physiol Biochem.

[32] Victor N, Ivy A, Jiang BH, Agani FH (2006). Involvement of HIF-1 in invasion of Mum2B uveal melanoma cells. Clin Exp Metastasis.

[33] Bernstein C, Holubec H, Bhattacharyya AK, Nguyen H, Payne CM, Zaitlin B (2011). Carcinogenicity of deoxycholate, a secondary bile acid. Arch Toxicol.

[34] Ajouz H, Mukherji D, Shamseddine A (2014). Secondary bile acids: an underrecognized cause of colon cancer. World J Surg Oncol.

[35] Jenkins GJ, Cronin J, Alhamdani A, Rawat N, D’Souza F, Thomas T (2008). The bile acid deoxycholic acid has a non-linear dose response for DNA damage and possibly NF-kappaB activation in oesophageal cells, with a mechanism of action involving ROS. Mutagenesis.

[36] Kazumori H, Ishihara S, Rumi MA, Kadowaki Y, Kinoshita Y (2006). Bile acids directly augment caudal related homeobox gene Cdx2 expression in oesophageal keratinocytes in Barrett’s epithelium. Gut.

[37] Roman S, Petre A, Thepot A, Hautefeuille A, Scoazec JY, Mion F (2007). Downregulation of p63 upon exposure to bile salts and acid in normal and cancer esophageal cells in culture. Am J Physiol Gastrointest Liver Physiol.

[38] Louis P, Hold GL, Flint HJ (2014). The gut microbiota, bacterial metabolites and colorectal cancer. Nat Rev Microbiol.

[39] Verberne L, Bach-Faig A, Buckland G, Serra-Majem L (2010). Association between the Mediterranean diet and cancer risk: a review of observational studies. Nutr Cancer.

[40] Phelan, John P. “HIF-1 and Bile Acids.” Open Science Framework. 2016. osf.io/8xh2k.

